# Development of a risk prediction model to predict the risk of hospitalization due to exacerbated asthma among adult asthma patients in a lower middle-income country

**DOI:** 10.1186/s12890-023-02773-1

**Published:** 2023-12-06

**Authors:** Dhanusha Harshinie Punyadasa, Vindya Kumarapeli, Wijith Senaratne

**Affiliations:** 1 Wayamba University of Sri Lanka, Kuliyapitiya, Sri Lanka; 2 Directorate of Non-Communicable Diseases, Ministry of Health, Colombo, Sri Lanka; 3 National Hospital for Respiratory Diseases, Welisara, Sri Lanka

**Keywords:** Asthma, Exacerbation, Hospitalization, Risk prediction model, Validation, Lower middle-income country, Sri Lanka

## Abstract

**Background:**

Asthma patients experience higher rates of hospitalizations due to exacerbations leaving a considerable clinical and economic burden on the healthcare system. The use of a simple, risk prediction tool offers a low-cost mechanism to identify these high-risk asthma patients for specialized care. The study aimed to develop and validate a risk prediction model to identify high-risk asthma patients for hospitalization due to exacerbations.

**Methods:**

Hospital-based, case-control study was carried out among 466 asthma patients aged ≥ 20 years recruited from four tertiary care hospitals in a district of Sri Lanka to identify risk factors for asthma-related hospitalizations. Patients (*n* = 116) hospitalized due to an exacerbation with respiratory rate > 30/min, pulse rate > 120 bpm, O2 saturation (on air) < 90% on admission, selected consecutively from medical wards; controls (*n* = 350;1:3 ratio) randomly selected from asthma/medical clinics. Data was collected via a pre-tested Interviewer-Administered Questionnaire (IAQ). Logistic Regression (LR) analyses were performed to develop the model with consensus from an expert panel. A second case-control study was carried out to assess the criterion validity of the new model recruiting 158 cases and 101 controls from the same hospitals. Data was collected using an IAQ based on the newly developed risk prediction model.

**Results:**

The developed model consisted of ten predictors with an Area Under the Curve (AUC) of 0.83 (95% CI: 0.78 to 0.88, *P* < 0.001), sensitivity 69.0%, specificity 86.1%, positive predictive value (PPV) 88.6%, negative predictive value (NPV) 63.9%. Positive and negative likelihood ratios were 4.9 and 0.3, respectively.

**Conclusions:**

The newly developed model was proven valid to identify adult asthma patients who are at risk of hospitalization due to exacerbations. It is recommended as a simple, low-cost tool for identifying and prioritizing high-risk asthma patients for specialized care.

**Supplementary Information:**

The online version contains supplementary material available at 10.1186/s12890-023-02773-1.

## Introduction

Asthma is the most common chronic respiratory disease affecting people in both affluent and developing countries [1]. Around 1% of all DALYs lost worldwide are found to be due to asthma, which reflects the high prevalence and severity of the disease. The hospitalization rate due to asthma remains high across the world despite therapeutic advancements [2].

Asthma is one of the leading causes of hospitalizations in government hospitals in Sri Lanka over the past two decades. According to the Annual Health Bulletin 2019 Sri Lanka, asthma was the top among the main non-communicable diseases responsible for hospitalisations to government hospitals in Sri Lanka, claiming 177,225 live discharges and 569 deaths [3]. These hospital admissions are disruptive and unsettling to the Sri Lankan healthcare system with limited resources.

It is estimated that majority of expenses for asthma care are incurred by the patients with the most severe disease and this is mainly due to the high cost of asthma-related hospital admissions and medication [4]. Therefore, minimizing asthma-related hospitalizations has been identified as the key to reducing the overall cost of asthma care.

Identification and management of asthma patients who are at risk of hospitalization is vital to minimize asthma-related hospitalizations. Risk prediction is growing in importance in this regard, and risk prediction models have been used to screen patients who are likely to get admitted due to exacerbated asthma [5–7]. The goal is to prevent their future admissions by providing specialized health care or lifestyle modifications. These models are also used to estimate the risk entailed by individual patients, which in turn guides their clinical management [8]. Thus, risk prediction models have considerable potential to contribute to the decision-making process, in the clinical management of asthma patients.

According to studies done in developed countries, various factors have been identified as risk predictors for hospitalizations due to exacerbated asthma. However, there is little knowledge about these risk predictors in Sri Lankan asthma patients who remain susceptible to exacerbation, while receiving treatment for secondary prevention.

Several studies have developed risk stratification schemes for asthma patients, using large healthcare databases [9, 10]. However, the results of these studies have frequently been too complicated to use for risk prediction in local settings. None of the available validated risk models to predict asthma hospitalization originated from the Southeast Asian Region. There have not been any previous attempts to develop and validate a country-specific risk prediction model to identify high-risk asthma patients in Sri Lanka. In this background, the present study aimed to develop and validate a risk prediction model to identify high-risk asthma patients for hospitalization due to exacerbations.

## Methods

Development and validation of the risk prediction model were performed in a stepwise manner (Supplementary Fig. 1).

### Identification of the risk factors for hospitalizations due to exacerbated asthma

As the first step, an unmatched case-control study was conducted from October 2018 to December 2018 in the Gampaha districts of Sri Lanka to identify the risk factors for hospitalizations due to exacerbated asthma. This study included 466 clinically confirmed asthma patients aged ≥ 20 years (116 cases and 350 controls) recruited from four tertiary care hospitals in the district.

The sample size was calculated using the formula for unmatched case-control studies with multiple controls per a case [11]. The sample size was calculated separately for each of the selected risk factors for asthma hospitalization and the risk factor having the largest sample size derived was used to calculate the sample. The corresponding risk factor for asthma hospitalization was ‘current smoking’ with an odd ratio of 1.86 and 34% of clinical asthma patients being exposed to this risk factor [12]. Case to control ratio was taken as 1:3 and a 5% for non-response rate was added to the calculated sample size.

Patients who have been diagnosed with asthma for more than 1 year and are presently hospitalized due to an exacerbation with an on-admission respiratory rate of > 30/min, pulse rate of > 120 bpm, (on air) O2 saturation of < 90% and required both regular nebulization and systemic steroids on admission were selected as cases (*n* = 116), consecutively from the medical wards of the selected hospitals.

Patients who have been diagnosed with asthma for more than 1 year, without any hospitalizations for an exacerbation during the past year were selected as controls (*n* = 350; 1:3ratio), randomly from asthma/medical clinics of the same hospitals. Patients diagnosed with other chronic respiratory diseases, tuberculosis, or heart failure in addition to asthma, patients who have been admitted to the private sector for an exacerbation, patients who have taken treatment in the emergency treatment unit and discharged for an exacerbation, patients who have been advised to get admitted for an exacerbation but refused during the past year were excluded as controls.

Information on asthma control, asthma co-morbidities, risk behaviours, and physiological and sociodemographic factors were obtained using an interviewer-administered questionnaire with verification through medical records when appropriate.

### Selection of predictors for the risk prediction model

We selected candidate predictors to be included in the risk prediction model by two methods as described below.

#### Method 1: statistical method

The variables that emerged as risk factors in the bivariate analysis of the case-control study with statistical significance at *p* < 0.05 were selected as candidate predictors for the Multiple Logistic Regression (MLR) model. These variables include age, education level, monthly income, having Diabetes Mellitus, having symptomatic Gastroesophageal Reflux Disease (GORD), use of Asprin, use of ACE inhibitors, having first degree relatives with asthma, having ever smoked, current smoker, number of pack years smoked, worked with solvents, exposure to traffic, exposure to secondhanded smoke, previous hospitalizations due to exacerbations, ever intubated or given Intensive Care Unit (ICU) care, level of asthma control, asthma treatment step, taking more than 200 doses of relievers per month, and Body Mass Index (BMI) category of patients (Supplementary Table 1). To improve the model performance and to reduce the risk of false positive findings, the Evets Per Variable (EPV) rule of thumb was applied in shortlisting the candidate predictors. This rule recommends that at least 10 individuals need to have developed the outcome of interest for every predictor variable to be included in the model [13]. Therefore, candidate predictors were reduced in relation to their frequency of the outcome. Out of 20 variables that showed significant associations with asthma hospitalization in the bivariate analysis at the statistical significance of *p* < 0.05, only 15 variables were selected as candidate predictors for the MLR analysis as the other five variables had cell counts of less than 10 among the exposed group.

#### Method 2: consensus of a panel of experts

The knowledge of experts in the field was also obtained to select candidate predictors for the model. The experts were provided with information on the factors that were found to be significant in the bivariate analysis of the case control study. They were also provided with the information on risk predictors that have been used in other risk prediction models to estimate the risk of asthma hospitalization, found in literature. The experts were requested to indicate their decision on candidate predictor that should be included to the model on a predesigned format. The completed formats were analysed and the factors that were agreed upon by 60% of the panel were selected as final predictors to be included into the model.

It was decided to eliminate two variables (exposure to second-hand smoke and exposure to traffic) from the list of candidate predictors by the panel of experts. Since exposure to second-hand smoke and exposure to traffic were measured only in relation to the household environment of the patients, the panel of experts suggested that it was not a comprehensive measurement of those risk predictors which could lead to biased risk estimation.

According to the analysis of expert opinion, no predictors were selected as additional predictors to be included in the MLR model.

### Development of the risk prediction model

MLR analyses by backward elimination method were performed to develop the model to predict the individual risk of an adult asthma patient for hospitalization due to exacerbation.

Out of the thirteen candidate predictors included in the MLR analysis, only ten variables remained significant in the final LR model (Supplementary Table 2). Adjusted predictors for hospitalization due to exacerbated asthma using multivariate analysis were age ≥ 60 years, education up to General Certificate of Education Ordinary Level (GCE O/L) or less, having Diabetes Mellitus, family history of asthma, having ever smoked, ever intubated/given ICU care, having previous hospitalizations due to asthma exacerbations, having uncontrolled asthma, having GORD and BMI ≥ 25 kg/m2. None of the predictor variables in the model had a standard error of more than 2.0, which indicated that there was no evidence of multi-collinearity between predictor variables.

### Assignment of ‘weighted scores’ to the predictors of the risk prediction model

The risk prediction model was designed as a formula where each risk predictor in the model was assigned a value(score) which depicts its relative contribution to predicting the risk of hospitalization due to exacerbated asthma. This simplified score was based on the original scale of the regression coefficients for each predictor in the final multivariable model.

The predictors selected for the final model were given their regression coefficients (Log odds) as weighted scores, rounded up to have no decimals. These weighted scores were summated into a single summary score to predict the individual risk of an asthma patient.

### Refining of the risk prediction model

Receiver Operator Characteristic (ROC) curve analysis was performed to refine the model. Discrimination of the model was tested by depicting the ROC curve and calculating the AUC using the validation sample described below. A summary risk score was derived for each case and control of the validation sample, by adding up all the individual weighted scores of the selected predictors of the risk prediction model. The predictors were included in the model one after another and for each of the models that were generated, ROC curve analysis and AUC analyses were performed. The predictor combination which gave the highest AUC was selected as the predictor combination of the final model.

### Validation of the risk prediction model

The criterion validity of the refined risk prediction model was assessed by conducting a validation study from May 2019 to June 2019 using an independent case-control design. The criterion validity of the model was assessed by comparing the hospitalization status of each asthma patient with the presence or absence of risk estimated by the risk prediction model.

The study population comprised asthma patients aged ≥ 20 years residing in the same district where the first case-control study was conducted, and who have been diagnosed with Asthma for more than 1 year with their diagnosis reviewed and reconfirmed by the Respiratory Physician or the General Physician. Cases were defined as those who were currently hospitalized for an exacerbation with respiratory rate > 30/min, Pulse rate > 120 bpm, O2 Saturation (on air) < 90% during the admission and required both regular nebulization and systemic steroids on admission.

A control was defined as a person, who has been diagnosed with asthma for more than 1 year, without any hospitalizations for an exacerbation during the past year and has been residing in the district of Gampaha for a minimum period of 1 year prior to the commencement of the study. Patients diagnosed with other chronic respiratory diseases, tuberculosis, or heart failure in addition to asthma, patients who have been admitted to the private sector for an exacerbation, patients who have taken treatment in the emergency treatment unit and discharged for an exacerbation, patients who have been advised to get admitted for an exacerbation but refused during the past year were excluded as controls. The cases were selected consecutively from the medical wards of all tertiary care hospitals in the district, while the controls were selected randomly from the medical clinics of the same hospitals.

In calculating the sample size, the minimum value of specificity and sensitivity found in the literature was taken as the anticipated population prevalence (P) to obtain the maximum sample size. The minimum specificity and sensitivity of risk prediction models for asthma hospitalization found in the literature were 84% [9] and 30% [10] respectively. A precision level of 12% was allowed with a confidence level of 90%. The minimal sample size required for cases was 158 and the minimal sample size required for controls was 101.

According to the score given for each of the predictors in the risk prediction model, a total risk score was derived for cases and controls by adding all the individual weighted scores.

ROC curve was plotted against true positive (sensitivity) and false-positive(1-specificity) rates to select the best cut-off point to categorize each participant into ‘at-risk’ or ‘not at-risk’ of hospitalization due to asthma. The test variable of the ROC curve was the summary score obtained for each individual while the state variable was the presence or absence of asthma hospitalization. The optimal cut-off value was obtained by the maximum length from the AUC to the diagonal line. ‘At-risk’ or ‘not at-risk’ categories specified by the risk prediction model were tested, against the true presence (cases) or absence (controls) of hospitalization due to asthma.

The indicators of the validity of the risk prediction model (sensitivity, specificity, PPV, NPV and likelihood ratios) were estimated based on the cut-off value.

Information on the risk predictors was obtained by a pre-tested structured IAQ developed based on the newly developed risk prediction model. IAQ was administered by three trained medical officers and informed and written consent was obtained from all study participants. The study was approved by the Ethics Review Committee of the Faculty of Medicine, University of Colombo, Sri Lanka.

A provision was given in the IAQ itself to indicate the score assigned to each risk predictor and the total risk score to facilitate the estimation of the total score for each patient at the end of administering the tool. Each risk predictor in the model was given its regression coefficients (Log odds) as its weighted score, rounded up to have no decimals. The reference level was assigned a score of zero.

## Results

All selected cases (*n* = 158) and controls (*n* = 101) participated in the validation study giving a response rate of 100%. 64% of asthma patients were females while 87% were Sinhalese and 62% were Buddhists. 60% were found to be unemployed. A statistically significant difference was observed between the development sample and validation sample in the following predictor variables; Educated ≤ GCE O/L (*p* < 0.05), previous hospitalizations due to exacerbations (*p* < 0.001) and having uncontrolled asthma (*p* < 0.05) (Supplementary Table 3).

**Fig. 1:**
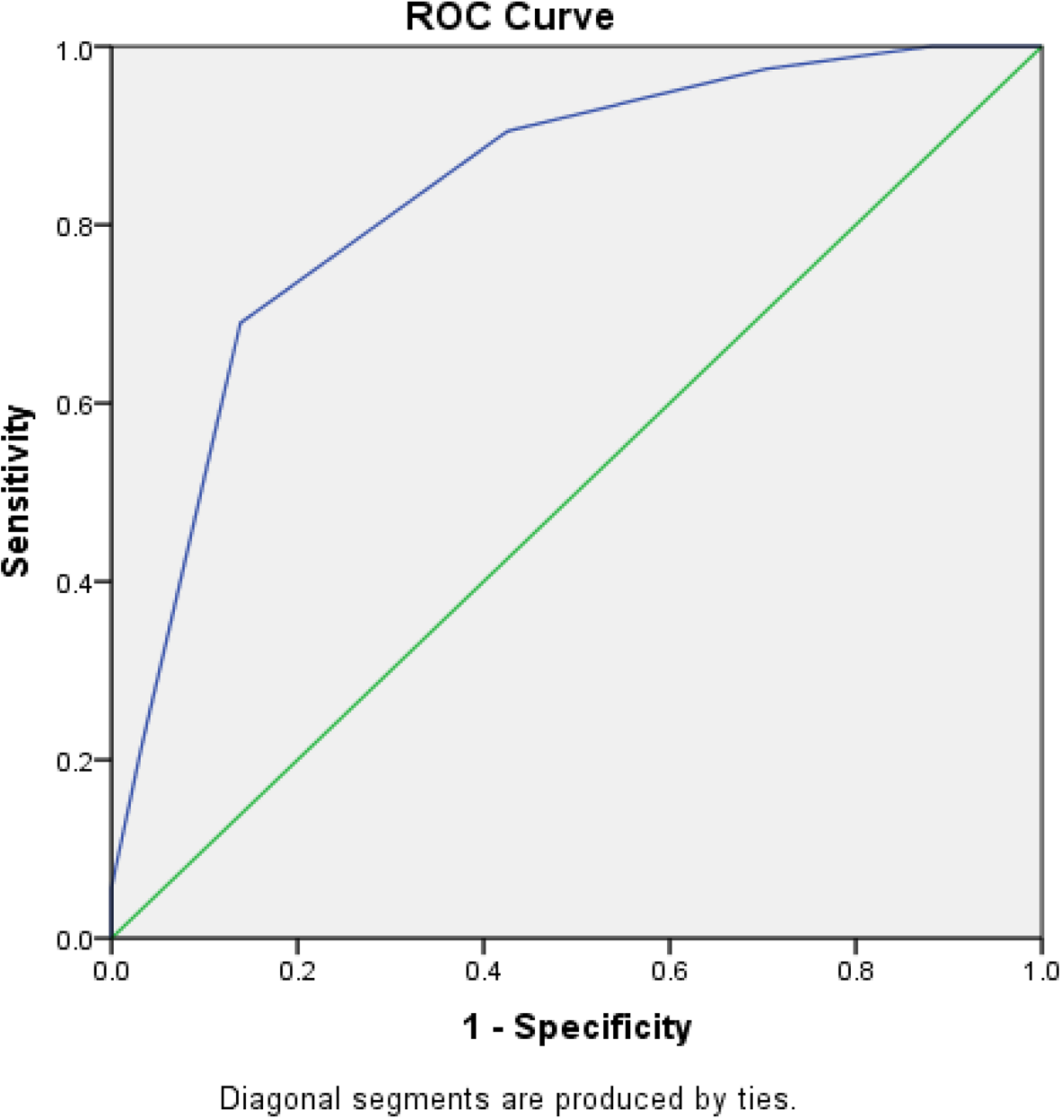
ROC curve for summary risk scores against the presence or absence of asthma hospitalization among study participants

The best model derived from the ROC analysis consisted of ten predictors giving the highest AUC of 0.83 (95% CI = 0.78 -0.88). Table 1 shows the best-performing risk prediction model with the risk predictors and their scores. Figure 1 shows the ROC analysis of the model. The AUC was statistically significant (*p* < 0.001) indicating a good model performance where 83% of the variability of the asthma hospitalization is explained by the summary risk score.

**Table 1:** Predictor variables in the best performing risk prediction model and their weighted scores

**Predictor variable**	**β coefficient**	**Assigned score**
Age ≥ 60 years	0.910	1
Educated ≤ G.C.E. O/L	0.719	1
Having Diabetes Mellitus	0.609	1
Family history of Asthma	0.625	1
Ever smoked	1.022	1
Ever intubated/ given ICU care	1.194	1
Previous hospitalizations due to exacerbations	1.619	2
Uncontrolled asthma	1.232	1
Having symptomatic GORD	1.029	1
BMI ≥ 25 kg/m^2^	0.889	1
**Total score**		**11**

*GCE O/L* General Certificate of Education Ordinary Level, *ICU* Intensive Care Unit, *GORD* Gastro Oesophageal Reflux Disease, *BMI* Body Mass Index

The summary risk score of the risk prediction model ranged from 1 to 11. The shortest distance (d^2^) in the ROC curve was 0.1154 which corresponded with the summary risk score of 4.5. This indicated that summary risk score of 4.5 is the optimal cut-off value to categorize each participant into ‘at risk’ or ‘not at risk’ of getting hospitalized due to exacerbated asthma.

At the 4.5 cut-off value, the model gave a sensitivity of 69.0% (95% CI 61.7%-76.2%), a specificity of 86.1% (95% CI 79.4%-92.8%), a positive predictive value of 88.6% (95% CI 79.4%-92.8%) and a negative predictive value of 63.9% (95% CI 55.9%-72.0%). The calculated likelihood ratios were LR+: 4.9 (95% CI 3.0–8.1) and LR-: 0.3(95% CI 0.2–0.4).

## Discussion

We aimed to develop a simple, low-cost, risk prediction tool that would be applicable to primary healthcare settings to identify at-risk asthma patients for hospitalization. The model was developed incorporating simple, measurable risk predictors that can be easily obtained from the history and examination of patients. This enabled application of the model in low-resource settings at the first contact care level. A simple ten-question interviewer-administered tool was designed to obtain information on risk predictors. A simple risk score was given to each asthma patient by this tool, which would allow clinicians to stratify patients for proactive management within the existing busy clinic setups.

Existing risk prediction models on asthma hospitalizations are either applicable to specialized populations [9, 14, 15], predictions are purely based on literature and statistical methods [10, 16] or are designed to predict different outcomes such as hospital readmissions [17] and emergency department visits [15]. Furthermore, the predictive performance of these models has often not been assessed beyond the population from which they were derived [9, 14, 15].

The present risk prediction model was primarily based on adjusted risk factors for asthma-related hospitalizations derived from a case-control study conducted among adult asthma patients in four local healthcare settings. Therefore, this model is more valid for local settings to apply to adult asthma patients. In identifying candidate predictors for the model, the opinion of experts in the field was also considered in addition to the statistical method. This allowed us to develop a more comprehensive and clinically relevant model. As prediction is all about estimation rather than testing of risk factors, it is recommended to include clinically relevant and reliable predictors for a risk prediction model [18].

Most of the asthma-specific risk prediction tools have utilized a lot of clinical information as predictors [19, 20] which would be costly in obtaining at a large scale. Other tools have incorporated subtle physical findings like accessory muscle use [20], spirometry [16] or peak expiratory flow rate variability [7, 10] which may reduce the feasibility of the tool and thereby the acceptance in the routine clinical practice. The ability to verify all risk predictors in the present model from a short history and examination is an advantage in the utilization of this model even in busy clinic settings.

It is recommended that a valid, reliable measurement of risk predictor is vital in risk assessment, as an inaccurate measurement of a risk predictor could lead to biased risk estimation [21]. Thus, ‘exposure to traffic’ and ‘exposure to second-hand smoke’ were not incorporated as risk predictors in the present model, even though they came as significant risk factors in the MLR analysis, considering the unreliability in assessing those predictors from the history. Risk predictors contained in the present risk prediction model such as age, education level, smoking status, ever admitted for asthma and intubation for asthma have also been identified by the previously published risk prediction models for asthma morbidity [16, 22–24]. Other risk predictors in the model; having Diabetes Mellitus [25], symptomatic GORD [26], family history of asthma [27], uncontrolled asthma [28] and obesity [29] have also been previously evaluated as risk factors for adverse asthma outcomes.

There were only a few studies which have assessed the discriminative power of risk prediction models related to hospitalization among adult asthma patients. The present model achieved a higher AUC of 0.83 (95% CI = 0.78–0.88) compared to other models validated [10, 16], indicating good discriminative power to differentiate between at-risk and not at-risk asthma patients for hospitalizations due to exacerbations.

The most acceptable cut-off level for the model was decided by the best trade-off between false negatives and false positives. If a high cut-off level was selected, it would increase the model specificity. However, at the same time it would decrease the sensitivity and in turn, would miss more at-risk asthma patients. It was considered that a more sensitive screening tool would be beneficial to detect high-risk asthma patients since asthma-related hospitalizations impose a high economic burden on the healthcare systems.

By setting the cut-off threshold at 4.5, our model was able to capture 69% of the asthma patients who will incur future hospitalizations due to exacerbations. Most of the prior models [9, 16, 30] reported to have sensitivity lower than the present model. These models have been developed commonly by using large healthcare databases and their predictor variables are limited to variables that can be obtained from those databases.

A case-control study design was adopted to evaluate the criterion validity of the present model considering the limitation of time and logistics. However, the ideal study design recommended for this purpose is a follow-up study where high-risk asthma patients should be followed up, to see whether they get hospitalized due to exacerbations as predicted by the risk prediction model [31].

The hospitalization status of these asthma patients was confirmed clinically using an admission criterion supported by clinic records. Verification bias is a possibility in this method, as there was no such gold standard test that can be applied to all participants to confirm their status of hospitalization. But this has been minimized by strictly adhering to admission criteria in selecting cases, and by reviewing and reconfirming their admission status by a clinician at both clinic and ward settings.

## Conclusions

The newly developed risk prediction model consisting of ten risk predictors was proven to be valid in identifying asthma patients aged 20 years and above who are at risk of hospitalization due to exacerbations in a semi urban population in Sri Lanka. It owned many features of a model that can be easily administered in low-resource primary health care settings. The simple risk score given to each asthma patient by this tool would enable clinicians to stratify asthma patients for proactive management within busy clinical settings.

### Supplementary Information

**Additional file 1: Supplementary Figure 1.** Steps in the development of the risk prediction model for hospitalizations due to exacerbated asthma.

**Additional file 2: Supplementary Table 1.** Significant risk factors for hospitalization due to exacerbated asthma among adult asthma patients via bivariate analysis in comparison with controls (Level of significance = 0.05). **Supplementary Table 2.** Model fitting information of the final MLR model with selected predictors. **Supplementary Table 3.** Distribution of the predictors among development sample and the external validation sample.

## Data Availability

The datasets used and/or analyzed during the current study are available from the corresponding author on reasonable request.

## Abbreviations

IAQ: Interviewer Administered Questionnaire
AUC: Area Under the Curve
PPV: Positive Predictive Value
NPV: Negative Predictive Value
DALY: Disability Adjusted Life Years
MLR: Multiple Logistic Regression
EPV: Events Per Variable
LR: Logistic Regression
GCE O/L: General Certificate of Examination Ordinary Level
ICU: Intensive Care Unit
GORD: Gastroesophageal Reflux Disease
BMI: Body Mass Index
ROC: Receiver Operator Curve
CI: Confidence Interval
LR+: Positive Likelihood Ratio
LR-: Negative Likelihood Ratio
