# Supplementary material for: Development of a risk prediction model to predict the risk of hospitalization due to exacerbated asthma among adult asthma patients in a lower middle-income country
Source: BMC Pulm Med. 2023 Dec 6;23:491. doi: 10.1186/s12890-023-02773-1 (PMC10698957; doi:10.1186/s12890-023-02773-1)
Supplement: Supplementary file 1 — Additional file 1: Supplementary Figure 1. Steps in the development of the risk prediction model for hospitalizations due to exacerbated asthma. [file 12890_2023_2773_MOESM1_ESM.docx]

**Final risk prediction model with 10 risk predictors**

**Refine of risk prediction model with ROC analysis**

**Assignment of weighted scores to the predictors of the risk prediction model**

**Identification of risk predictors**

Consensus from a panel of experts

**Controls (n = 350; 1:3 ratio)**

Asthma patients,

- who has been diagnosed of Asthma for more than 1 year,
- **without any hospitalizations for an exacerbation during the past year**.

selected randomly from asthma/medical clinics

**Hospital based case control study**

Among **466** asthma patients aged ≥20 years

in four tertiary care hospitals in Gampaha district

**Cases (n=116)**

asthma patients hospitalized due to an exacerbation.

- respiratory rate >30/min
- pulse rate >120 bpm
- O2 saturation (on air) < 90% on admission

selected consecutively from medical wards

Logistic regression analysis

**Supplementary Figure 1; Steps in the development of the risk prediction model for hospitalizations due to exacerbated asthma.**

ROC- Receiver Operator Characteristic.
