# Supplementary material for: Development of a risk prediction model to predict the risk of hospitalization due to exacerbated asthma among adult asthma patients in a lower middle-income country
Source: BMC Pulm Med. 2023 Dec 6;23:491. doi: 10.1186/s12890-023-02773-1 (PMC10698957; doi:10.1186/s12890-023-02773-1)
Supplement: Supplementary file 2 — Additional file 2: Supplementary Table 1. Significant risk factors for hospitalization due to exacerbated asthma among adult asthma patients via bivariate analysis in comparison with controls (Level of significance = 0.05). Supplementary Table 2. Model fitting information of the final MLR model with selected predictors. Supplementary Table 3. Distribution of the predictors among development sample and the external validation sample. [file 12890_2023_2773_MOESM2_ESM.docx]

| **Variable** | **Categories** | | **Un-adjusted OR** | | **95% CI** | | | | **P** |
| --- | --- | --- | --- | --- | --- | --- | --- | --- | --- |
| Age group | ≥ 60 years | | 2.0 | | 1.3–3.2 | | | | 0.002 |
| Education level | ≤ G.C.E. O/Level | | 2.1 | | 1.1-4.2 | | | | 0.025 |
| Monthly income | ≤ Rs 20,000 | | 2.0 | | 1.3-3.2 | | | | 0.001 |
| Having Diabetes Mellitus | Yes | | 1.9 | | 1.1-3.2 | | | | 0.008 |
| Having symptomatic GORD | Yes | | 2.2 | | 1.3-3.6 | | | | 0.001 |
| Use of Asprin | Yes | | 4.4 | | 1.3-14.3 | | | | 0.013 |
| Use of ACE inhibitors | Yes | | 5.8 | | 1.9-17.0 | | | | 0.001 |
| First degree relative with asthma | Yes | | 1.7 | | 1.1-2.7 | | | | 0.009 |
| Ever smoked | Yes | | 2.7 | | 1.6-4.7 | | | | <0.001 |
| Current smoker  Number of pack years smoked | Yes  ≥20 pack years | | 6.3  3.2 | | 1.5-25.8  1.3-7.9 | | | | 0.003  0.008 |
| Worked with solvents | Yes | | 7.8 | | 1.5-41.2 | | | | 0.012 |
| Exposure to traffic | Yes | | 2.4 | | 1.3-4.7 | | | | 0.005 |
| Exposure to secondhand smoke | Yes | | 1.7 | | 1.0-2.9 | | | | 0.040 |
| Previous hospitalizations due to exacerbations | | Yes | 4.8 | | 2.6-8.8 | | | <0.001 | |
| Ever intubated/given ICU care | | Yes | 3.2 | | 1.4-7.5 | | | 0.003 | |
| Level of asthma control | | Not controlled | 3.8 | | 2.0-7.3 | | | <0.001 | |
| Asthma treatment step | | On high dose steroids | 3.0 | | 1.9-4.7 | | | <0.001 | |
| Takes > 200 doses of relievers per month | | Yes | 5.1 | | 1.6-16.0 | | | 0.005 | |
| BMI category | | ≥25 kg/m^2^ | 2.2 | | | 1.4-3.4 | | | <0.001 |
|  | |  | |  | | |  | |  |

**Supplementary Table 1: Significant risk factors for hospitalization due to exacerbated asthma among adult asthma patients via bivariate analysis in comparison with controls (Level of significance =0.05)**

| **Predictor variable** | **ᵦ** | **SE (ᵦ)** | **Exp (ᵦ)** | **95% CI for exp (ᵦ)** | | | **Significance** |
| --- | --- | --- | --- | --- | --- | --- | --- |
|  |  |  |  | **Lower** | | **Upper** |  |
| Age ≥ 60 years | 0.910 | 0.278 | 2.48 | 1.44 | | 4.28 | 0.001 |
| Educated ≤ G.C.E. O/Level | 0.719 | 0.328 | 2.05 | 1.07 | | 3.90 | 0.028 |
| Having Diabetes Mellitus | 0.609 | 0.301 | 1.83 | 1.01 | | 3.31 | 0.043 |
| Family history of asthma | 0.625 | 0.262 | 1.86 | 1.11 | | 3.12 | 0.017 |
| Ever smoked | 1.022 | 0.323 | 2.78 | 1.47 | | 5.23 | 0.002 |
| Ever intubated/ given ICU care | 1.194 | 0.404 | 3.30 | 1.49 | | 7.29 | 0.003 |
| Previous hospitalizations due to exacerbations | 1.619 | 0.358 | 5.04 | 2.50 | | 10.18 | 0.000 |
| Uncontrolled asthma | 1.232 | 0.363 | 3.42 | 1.68 | | 6.98 | 0.001 |
| Having symptomatic GORD | 1.029 | 0.301 | 2.79 | 1.55 | | 5.04 | 0.001 |
| BMI ≥25 kg/m^2^ | 0.889 | 0.259 | 2.43 | 1.46 | | 4.04 | 0.001 |
| Constant | -5.346 | 0.628 | 0.005 |  |  | | 0.000 |

Supplementary Table 2: Model fitting information of the final MLR model with selected predictors

ᵦ-regression coefficient

SE (ᵦ) –standard error of ᵦ

df –degree of freedom

**Supplementary Table 3: Distribution of the predictors among development sample and the external validation sample**

| **Predictor** | **Development sample (n=443)** | | **External validation sample**  **(n=259)** | | **(χ^2^)** | **Statistical significance**  **(p)** | **“Number of events per predictor” in the external validation sample** |
| --- | --- | --- | --- | --- | --- | --- | --- |
|  | **No** | **%** | **No** | **%** |  |  |  |
| Age ≥ 60 years | 249 | 56.2 | 157 | 60.6 | 1.30 | 0.25 | 110 |
| Educated ≤ G.C.E. O/Level | 368 | 83.1 | 159 | 61.4 | 41.04 | 0.01 | 98 |
| Having Diabetes Mellitus | 90 | 20.3 | 67 | 25.9 | 2.90 | 0.08 | 47 |
| Family history of asthma | 202 | 45.6 | 123 | 47.5 | 0.23 | 0.62 | 87 |
| Ever smoked | 67 | 15.1 | 43 | 16.6 | 0.27 | 0.60 | 36 |
| Ever intubated/ given ICU care | 24 | 5.4 | 21 | 8.1 | 3.11 | 0.08 | 16 |
| Previous hospitalizations due to exacerbations | 50 | 11.3 | 58 | 22.3 | 15.43 | <0.001 | 50 |
| Uncontrolled asthma | 324 | 73.1 | 216 | 83.4 | 9.69 | 0.001 | 149 |
| Having symptomatic GORD | 95 | 21.4 | 70 | 27.0 | 2.88 | 0.08 | 43 |
| BMI ≥25 kg/m^2^ | 189 | 43.0 | 115 | 44.4. | 0.20 | 0.65 | 81 |
